# Substitution of Arginine 120 in Human D-Amino Acid Oxidase Favors FAD-Binding and Nuclear Mistargeting

**DOI:** 10.3389/fmolb.2019.00125

**Published:** 2019-11-12

**Authors:** Giulia Murtas, Silvia Sacchi, Loredano Pollegioni

**Affiliations:** Dipartimento di Biotecnologie e Scienze della Vita, Università degli Studi dell'Insubria, Varese, Italy

**Keywords:** flavoprotein, D-serine, mistargeting, structure-function relationships, peroxisome

## Abstract

The peroxisomal enzyme human D-amino acid oxidase (hDAAO) is attracting attention owing to its role in degrading D-serine, the main co-agonist of N-methyl D-aspartate receptors in brain, and its involvement in brain functions and diseases. Here, we focused on arginine 120, a residue located at the protein interface, 20 Å from the assumed second ligand-binding site, showing a different orientation of the side chain in the hDAAO-benzoate complex, and corresponding to Ser119 in rat DAAO, which is part of a putative nuclear translocation signal (NTS). By substituting Arg120 in hDAAO with a glutamate (to mimic the active NTS) or a leucine (to eliminate the positive charge) the protein conformation, thermal stability, and kinetic properties are slightly altered, while the dimeric structure and the ligand-binding properties are unchanged. The most relevant alteration in Arg120 variants is the strongest interaction with FAD. Nevertheless, the activity assayed at low D-serine and FAD concentrations (resembling physiological conditions) was quite similar for wild-type and Arg120 hDAAO variants. These results resemble the ones obtained substituting another residue located at the interface region (i.e., the W209R variant), indicating that substitutions at the monomer-monomer interface mainly affects the FAD binding in hDAAO. Indeed, U87 glioblastoma cells transiently transfected for hDAAO variants show that substitution of Arg120 favors mistargeting: the increase in cytosolic localization observed for the variants promotes nuclear targeting, especially for the R120E hDAAO, without affecting cell viability. Notably, mistargeting to the nucleus is an innate process as it is apparent for the wild-type hDAAO, too: whether such a process is related to specific pathologic processes is still unknown.

## Introduction

Human D-amino acid oxidase (hDAAO, EC 1.4.3.3) is a FAD-dependent enzyme, which catalyzes the oxidative deamination of neutral D-amino acids into the corresponding α-keto acids, ammonia, and hydrogen peroxide (Pollegioni et al., 2007; Sacchi et al., 2012; Murtas et al., 2017). In the brain, the main physiological substrate of this flavoenzyme is D-serine (D-Ser). D-Ser binds the glycine site of the N-methyl-D-aspartate type of the glutamate receptors (NMDAr) regulating its activity (Mothet et al., 2000; Li et al., 2013; Le Bail et al., 2015). A number of experimental results suggests that dysregulation of processes tuning D-Ser concentrations and thus of NMDAr-dependent neurotransmission is involved in the mechanisms that concur to trigger various diseases such as amyotrophic lateral sclerosis, Alzheimer's disease, schizophrenia, etc. (Chumakov et al., 2002; Verrall et al., 2007; Billard, 2008; Madeira et al., 2008; Wolosker et al., 2008; Collingridge et al., 2013; Curcio et al., 2013; Murtas et al., 2017). Astrocytes express DAAO, the enzyme responsible for D-Ser degradation and thus ultimately modulate NMDAr-dependent physiological functions (Wolosker, 2011).

During the past few years, hDAAO structural-functional relationships have been studied in depth using the recombinant protein produced in *E. coli* (Kawazoe et al., 2006; Molla et al., 2006; Pollegioni et al., 2007; Caldinelli et al., 2009, 2010; Romano et al., 2009; Sacchi et al., 2012; Murtas et al., 2017) and cell lines overexpressing the enzyme (Sacchi et al., 2008, 2011; Li et al., 2013). Nevertheless, several aspects in the modulation of the enzyme activity remain elusive (Pollegioni et al., 2018). DAAO is known as a peroxisomal enzyme (Moreno et al., 1999; Sacchi et al., 2008; Cappelletti et al., 2014) that is targeted to this organelle due to the C-terminal PTS1 sequence. In peroxisomes, the cytotoxic product hydrogen peroxide generated by the DAAO reaction is eliminated by specific enzymes, such as catalase. U87 glioblastoma cells transiently overexpressing hDAAO showed that the flavoenzyme is cytosolic before being delivered to peroxisomes: a time course of protein import was reported in Sacchi et al. (2008, 2011). Recent reports on rats demonstrated that DAAO is present both in cytosol and nuclei of proximal tubule epithelial cells following treatment with the drug propiverine (Luks et al., 2017a). In the nucleus, DAAO is ubiquitinated, sumoylated, and degraded through the nuclear proteasomal system (Luks et al., 2017a,b). The mistargeting of the enzyme could be due to the presence of an NTS (TPx sequence corresponding to 117T-P-S119) that, after phosphorylation of Ser119, might activate the translocation to the nucleus (Chuderland et al., 2008; Luks et al., 2017a). Ser119 of rat DAAO (rDAAO) corresponds to Arg120 in the hDAAO sequence. Notably, deletion of the C-terminal peroxisomal targeting signal 1 (PTS1) in both hDAAO and rDAAO resulted in a diffused nuclear and cytosolic distribution of the flavoenzyme (Luks et al., 2017b): nuclear localization was higher for rDAAO probably because a serine is phosphorylated more frequently than an arginine or was due to the different molecular masses of the two homologous enzymes (40 vs. 80 kDa for rDAAO and hDAAO, respectively). Substitution of the putative tripeptide for nuclear translocation with the EPE sequence fully abolished nuclear import, independently of the presence of the PTS1 signal (Luks et al., 2017b).

**Figure 1:**
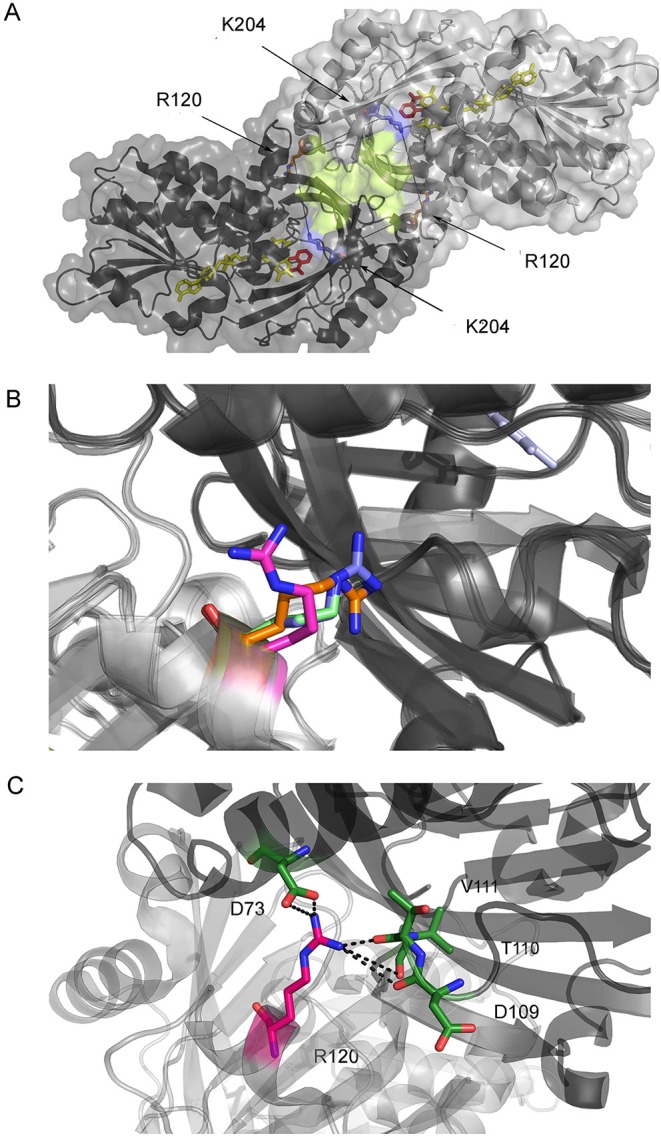
Structure of hDAAO. **(A)** Details of the monomer-monomer interface of hDAAO. The second putative benzoate binding site proposed by Kohiki et al. (2017) is depicted in yellow. **(B)** Alternative orientations of R120 in hDAAO in the free form (blue, pdb 2E48) and in complex with benzoate (magenta, pdb 2DU8), with imino-DOPA (green, pdb 2E82), and with imino-serine (orange, pdb 2E49). **(C)** Details of the interaction of R120 with residues of the facing subunit (the two protomers are depicted in a different color).

In hDAAO, the binding of an active site ligand facilitates FAD-apoprotein interaction to yield the active holoenzyme (Caldinelli et al., 2010; Murtas et al., 2017), this representing a main regulatory mechanism of its activity. R120 is located at the monomer-monomer interface (Figure 1A) and it has been also proposed to play a role in ligand binding. Binding of benzoate (an active site ligand) to hDAAO is a biphasic process (Murtas et al., 2017). Indeed, the FAD-binding to hDAAO apoprotein in the absence of an active site ligand is also biphasic, while a single phase of saturation is apparent in the presence of 70 μM benzoate (Murtas et al., 2017). For both FAD and benzoate binding no evidence of cooperativity between the first and second binding process was observed, which indicates the presence of two alternative protein conformations. A second benzoate binding site located in a cleft between the monomers was proposed (Kohiki et al., 2017): it is constituted by the residues belonging to the loop between β-strands 8 and 9 (residues 205-211) and those between β-strands 10 and 11 (residues 231-236); see Figure 1A. The overlay of the solved structures of hDAAO (in detail, 2DU8 in complex with benzoate, 2E82 in complex with imino-DOPA, 2E49 in complex with imino-serine, and 2E48, the substrate-free holoenzyme) does not show any difference in the residues belonging to the putative second binding site and pinpoints the orientation of R120 in the structure of hDAAO in complex with benzoate (Figure 1B) as being the only difference. R120 is located at the protein interface at 20 Å from the residues of the second binding site (Figure 1A).

Owing to the relevant physiological and pathological role of hDAAO, we expressed and biochemically characterized two variants at position 120 generated to mimic the activation of the NTS (by modifying the TPx sequence) and to verify its involvement in ligand binding and protein functionality.

## Materials and Methods

### Preparation and Expression of hDAAO Variants in *Escherichia coli*

Mutagenesis reactions to prepare His-hDAAO R120E and R120L variants were performed on the pET11b-His-hDAAO wild-type expression plasmid (Molla et al., 2006) using the QuickChange site-directed mutagenesis kit (Stratagene, Santa Clara, CA, USA). Previous studies showed that the His-tag at the N-terminal end of hDAAO did not alter its activity or stability (Molla et al., 2006; Romano et al., 2009). Recombinant wild-type and R120 hDAAO variants were expressed in BL21 (DE3) Star *E. coli* cells and purified as reported in Molla et al. (2006) and Romano et al. (2009); 40 μM of free FAD was present during all purification steps. The final enzyme preparations were stored at −20°C in 20 mM Tris-HCl buffer, pH 8.0, 100 mM NaCl, 10% (v/v) glycerol, 40 μM FAD, and 5 mM 2-mercaptoethanol (added for long-term storage). Final enzyme preparations have a purify degree >95% as judged by SDS-PAGE analysis. The enzyme concentration was determined spectrophotometrically by using the extinction coefficient at 445 nm (12.2 mM^−1^ cm^−1^) (Molla et al., 2006).

### Activity Assay and Kinetic Measurements

DAAO activity was assayed with an oxygen electrode at pH 8.5, air saturation, and 25°C, using 28 mM D-alanine as the substrate in the presence of 0.2 mM FAD (Molla et al., 2000, 2006). The kinetic parameters were determined on different D-amino acids, employing increasing substrate concentrations (Molla et al., 2000, 2006). The apparent kinetic parameters were calculated using the initial reaction rate according to a Michaelis–Menten equation using the Kaleidagraph software (Synergy Software, Reading, PA, USA).

### Spectral Measurements

Ligand and FAD-binding to hDAAO were investigated by following the quenching of protein fluorescence (0.3 mg protein/mL) (Molla et al., 2000; Caldinelli et al., 2009; Murtas et al., 2017). Protein fluorescence spectra were recorded between 300 and 400 nm, with excitation at 280 nm using a Jasco FP-750 instrument (excitation slit of 10 nm; emission slit of 5 nm) in 10 mM potassium phosphate, pH 8.0, 1% (v/v) glycerol, 5 mM 2-mercaptoethanol, and 40 μM FAD.

Circular dichroism (CD) spectra were recorded in 10 mM Tris-HCl, pH 8.0, 1% (v/v) glycerol, and 40 μM FAD by using a Jasco J-815 spectropolarimeter and analyzed employing a dedicated software (Jasco Co., Cremella, Italy). Cell path length was 1 cm for measurements above 250 nm (at 0.4 mg protein/mL) and 0.1 cm for measurements in the 190- to 250-nm region (at 0.1 mg protein/mL) (Caldinelli et al., 2009, 2010).

Temperature-ramp experiments were performed in 10 mM potassium phosphate, pH 8.0, 1% glycerol, 5 mM 2-mercaptoethanol, and 40 μM FAD. A software-driven, Peltier-equipped fluorimeter was used to measure the fluorescence protein changes at 340 nm and Peltier-equipped CD spectropolarimeter to follow the CD signal at 220 nm: a temperature gradient of 0.5°C/min (Caldinelli et al., 2009, 2010).

### Oligomeric State

The molecular mass in solution of hDAAO variants (0.1–10 mg/mL protein) was investigated by means of gel-permeation chromatography on a Superdex 200 Increase column, using an Akta chromatographic system (GE Healthcare, Uppsala, Sweden), at room temperature and in 20 mM Tris–HCl, pH 8.5, 150 mM NaCl, 5% glycerol, 40 μM FAD, and 5 mM 2-mercaptoethanol.

### Cell Cultures and Transfection

Human U87 glioblastoma cells (ATCC) were cultured in DMEM supplemented with 10% fetal bovine serum, 1 mM sodium pyruvate, 2 mM L-glutamine, 1% non-essential amino acids, 1% penicillin/streptomycin, and 1% amphotericin B (Euroclone), at 37°C in 5% CO_2_. The cells were transfected either with the pEYFP-hDAAO-C3 construct (for the expression of the wild-type enzyme downstream of the enhanced yellow fluorescent protein, EYFP) (Sacchi et al., 2008, 2011), the pEYFP-hDAAO(R120L)-C3, or the pEYFP-hDAAO(R120E)-C3 expression vectors. The latter constructs were obtained by mutagenesis using the QuikChange Lightning Site-Directed Mutagenesis Kit (Agilent), and the following primers: 5′-ATTTCGGAAGCTGACCCCCGAGGAGCTGGATATGTTCCCAG-3′ and 5′-CTGGGAACATATCCAGCTCCTCGGGGGTCAGCTTCCGAAAT-3′ for the R120E substitution, and 5-TTCGGAAGCTGACCCCCTTAGAGCTGGATATGTTCC-3′ and 5′-GGAACATATCCAGCTCTAAGGGGGTCAGCTTCCGAA-3′ for the R120L substitution.

For transient transfection, 2 ×10^4^ cells were seeded into eight-well chamber slides (Sarstedt). At 24 h after seeding, cells were transfected with 2 μg plasmid DNA using a 4:1 ratio of FuGENE transfection reagent (Promega) to DNA. Then, 12.5 μL of transfection mix was added to 500 μL medium per well. Protein expression levels were monitored by using a fluorescence microscope (Olympus IX51) equipped with a FITC filter and by Western blot analysis using an anti-hDAAO antibody (Davids Biotechnologie) (Sacchi et al., 2008, 2011).

### Immunostaining and Confocal Microscopy

At 24, 48, and 72 h after transfection, cells were washed twice with PBS and fixed with ice-cold 4% p-formaldehyde and 4% sucrose for 10 min at room temperature. After fixation, cells were washed twice with PBS and blocked by incubation in PBS supplemented with 0.2% Triton X-100 and 4% horse serum for 30 min at room temperature. Peroxisomes were stained using rabbit polyclonal anti-PMP70 (peroxisomal membrane protein 70, Sigma), diluted 1:500 in PBS, 0.1% Triton X-100, and 4% horse serum overnight at 4°C; they were then washed twice in PBS and 1% horse serum and incubated for 1 h at room temperature (light protected) with goat anti-rabbit Dylight 550 secondary antibodies (Abcam) diluted 1:400 in PBS, 0.1% Triton X-100, and 1.5% horse serum. Cells were then extensively washed in PBS and nuclei were stained with DRAQ5™ (Thermo Scientific) and diluted 1:500 in PBS for 10 min at room temperature in the dark. Cells were washed three times with PBS and stored in PBS at 4°C until imaging.

Immunostained cells were imaged using an inverted laser scanning confocal microscope (TCS SP5, Leica Microsystems), equipped with a 63.0 ×1.25 NA plan apochromatic oil immersion objective. Confocal image stacks were acquired using the Leica TCS software with a sequential mode to avoid interference between each channel and without saturating any pixel. Cells in 10–15 non-magnified optical fields were analyzed for each condition (i.e., different EYFP-hDAAO expression constructs at different times after transfection). The fluorescence signal corresponding to the overexpressed EYFP-hDAAO variants was monitored by excitation at 514 nm (yellow channel); the peroxisomal and nuclear cellular compartments were imaged by excitation at 546 nm (red channel) and 647 nm (blue channel), respectively.

## Results

### Design of R120 hDAAO Variants

Analysis of the hDAAO primary sequence for nuclear localization (e.g., by NucPred or cNL Mapper) and for the presence of nuclear localization sequences (e.g., by Moseslab, NLSdb by RostLab, or SeqNLS) does not predict hDAAO targeting to the nucleus. A putative nuclear translocation signal for rDAAO (117T-P-S119) was recently proposed (Luks et al., 2017b): this might facilitate nuclear import through importin 7 after phosphorylation of such a sequence. In hDAAO, this tripeptide corresponds to the 118T-P-R120 sequence.

R120 belongs to α-helix 6 and is involved in electrostatic interactions with the carboxylic group of D73 (α-helix 3) and hydrogen-bonded to the backbone C = O groups of D109 (α-helix 5), T110, and V111 (loop between α-helix 5 and β-strand 4) located at the interface of the opposite monomer (Figure 1C). Indeed, and as stated in the Introduction section, the R120 side chain possesses two alternative orientations, one of which is observed in the benzoate-bound complex only. R120 is located at the monomer-monomer interface (Figure 1B); therefore, a connection between protomer interaction and the binding of the ligand and/or the cofactor is feasible.

Based on these considerations, and to shed light on benzoate binding, monomer-monomer interaction, and nuclear translocation, we designed two hDAAO variants differing at position 120 aimed at eliminating the positive charge (introducing a Leu) or at mimicking the negative charge of a phosphorylated serine present at the active NTS in rDAAO (introducing a glutamate). A serine at position 120 was not introduced since no evidence is reported of phosphorylation in hDAAO (Murtas et al., 2017).

### Biochemical Properties of R120E and R120L hDAAO Variants

hDAAO R120E and R120L variants were expressed in *E. coli* following the same conditions used for expressing the wild-type enzyme and were purified by HiTrap chelating chromatography as previously reported (Molla et al., 2006). The protein preparations have a purity degree of 95% and the overall purification yield is 2.5- to 4-fold lower than the wild-type hDAAO (Table 1).

**Table 1:** Comparison of selected properties of recombinant wild-type, R120E, and R120L hDAAO variants.

	**hDAAO**		
	**Wild-type^**a**^**	**R120E**	**R120L**
**Protein purification**			
Purified protein (mg/L fermentation broth)	7.0	2.8	1.8
Specific activity (U/mg protein)	12.0	21.0	17.6
Holoenzyme content (%)	80	100	100
**Thermal stability (T**_**M**_,**°C)**			
CD (at 220 nm)	57.0 ± 1.0	46.1 ± 0.1	47.6 ± 0.1
Protein fluorescence (at 340 nm)	57.7 ± 0.3	52.7 ± 0.1	54.2 ± 0.1

a *Molla et al. (2006) and Caldinelli et al. (2009)*.

**Figure 2:**
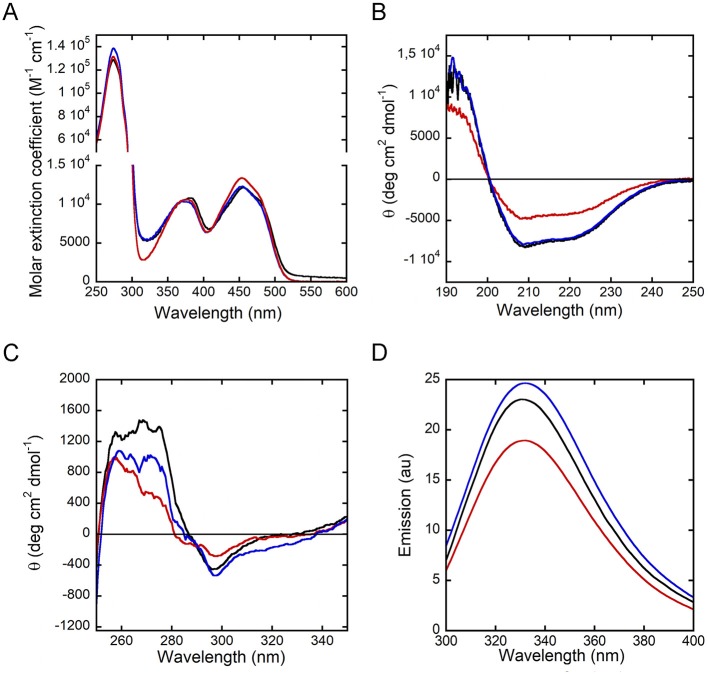
Spectral properties of hDAAO variants in the holoenzyme form. Wild-type (black), R120E (red) and R120L (blue) hDAAO variants. Comparison of: **(A)** UV-visible absorbance spectra in the oxidized form; **(B,C)** far-UV CD spectra (0.1 mg/mL, **B**), and near-UV CD spectra (0.4 mg/mL, **C**); **(D)** protein fluorescence spectra (0.3 mg/mL).

The R120E and R120L hDAAO variants show the typical absorbance spectrum of FAD-containing flavoenzymes in the oxidized form (Figure 2A): absorbance maxima at 448, 384, and 279 nm and an Abs_279nm_/Abs_448nm_ ratio of 10.4 and 11.2 for R120E and R120L variants, respectively, which are close to the value of 10.6 reported for the wild-type enzyme (Molla et al., 2006).

The far-UV CD spectra show a different signal for hDAAO R120E as compared to the wild-type enzyme and the R120L variant, indicating a lower α-helix content (Figure 2B). Concerning the tertiary structure, the near-UV CD spectra of hDAAO R120E and R120L are slightly altered in the 250- to 290-nm region, corresponding to the signal for aromatic residues, in comparison to the wild-type enzyme (Figure 2C). Accordingly, the protein fluorescence spectra for both of the variants at position 120 show a lower emission intensity of the peak at 330 nm, particularly for the R120E hDAAO (Figure 2D), strengthening the evidence that aromatic residue exposure has been altered.

Overall, the substitution of R120 results in a slight alteration in the conformation (secondary and tertiary structure) of hDAAO, which is most apparent for the R120E variant.

### Kinetic Properties

The activity assays performed in the absence of exogenous cofactor or the presence of an excess of FAD result in similar values indicating that purified R120E and R120L hDAAO are present in solution as holoenzyme, this being different from the wild-type enzyme, which is partially present in the apoprotein form in the absence of exogenous FAD (Table 1; see below). Notably, the R120E and R120L hDAAO variants show higher specific activity than the wild-type enzyme in the standard assay (on 28 mM D-alanine, Table 1).

The apparent kinetic parameters were calculated according to a Michaelis–Menten equation using the initial velocity values determined at increasing D-amino acid concentrations and a fixed O_2_ concentration (air saturation) at pH 8.5 and 25°C, in the presence of 0.2 mM FAD to avoid apoprotein formation for wild-type hDAAO. Three D-amino acids were used: D-Ala is the reference substrate for DAAOs (Pollegioni et al., 2007), D-Ser is considered the main substrate for hDAAO in the brain (based on the concentration and physiological role) (Sacchi et al., 2012; Murtas et al., 2017; Weatherly et al., 2017), and D-Cys is the putative preferred substrate in specific tissues (Shibuya et al., 2013; Murtas et al., 2017). Both variants show a 2-fold higher apparent kinetic efficiency (*k*_*cat*_/K_m_) for D-Ala than the wild-type hDAAO (due to a higher *k*_*cat*_; Table 2). On the other hand, using D-Ser as a substrate, all the enzyme variants show similar kinetic efficiency, although both apparent *k*_*cat*_ and K_m_ values are significantly higher in the R120-substituted variants (2.5- and 3.5-fold for the R120E and R120L, respectively; Table 2). An inhibition effect is observed at very high D-Cys concentration for both R120 variants (K_i_ > 200 mM), which is absent for wild-type hDAAO. Moreover, the kinetic efficiency of the R120L variant is 2-fold higher than the wild-type hDAAO on this substrate due to an increase in the apparent *k*_*cat*_ value.

**Table 2:** Comparison of the apparent kinetic properties of wild-type, R120E, and R120L hDAAO variants.

	**D-Ala**			**D-Ser**			**D-Cys**		
**hDAAO**	***k_***cat***_* (s^**−1**^)**	**K_**m**_ (mM)**	***k_***cat***_*/K_**m**_ (mM^**−1**^ s^**−1**^)**	***k_***cat***_* (s^**−1**^)**	**K_**m**_ (mM)**	***k_***cat***_*/K_**m**_ (mM^**−1**^ s^**−1**^)**	***k_***cat***_* (s^**−1**^)**	**K_**m**_ (mM)**	***k_***cat***_*/K_**m**_ (mM^**−1**^ s^**−1**^)**
wild-type^a^	5.2 ± 0.1	1.3 ± 0.2	4.0	3.0 ± 0.1	7.5 ± 0.5	0.4	8.6 ± 0.2	0.6 ± 0.1	14.6
R120E	12.6 ± 0.1	1.4 ± 0.1	8.9	8.5 ± 0.4	17.6 ± 3.5	0.5	9.9 ± 0.5	0.6 ± 0.1	16.5
R120L	17.3 ± 0.3	2.0 ± 0.3	8.5	11.3 ± 0.4	26.7 ± 4.5	0.4	15.6 ± 0.7	0.7 ± 0.1	22.6

*Conditions: air saturation, 25°C, pH 8.5*.

a *Molla et al. (2006) and Murtas et al. (2017)*.

**Figure 3:**
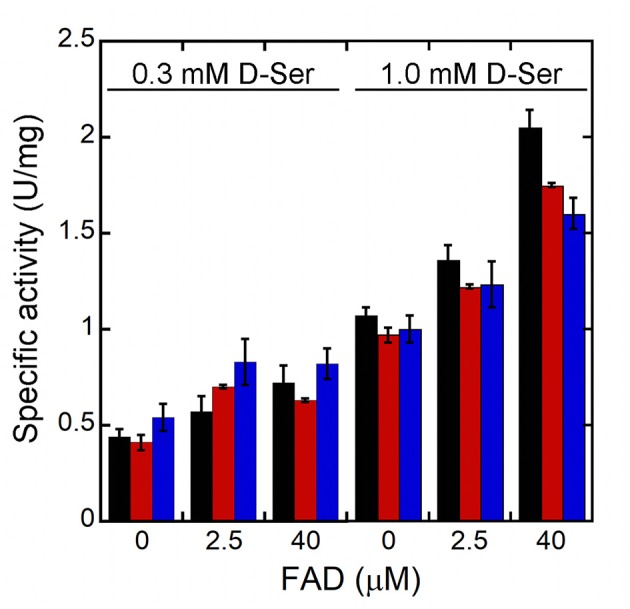
Effect of the concentration of the FAD on the activity of hDAAO variants at low substrate concentrations (resembling physiological conditions). Wild-type (black bars) R120E (red bars) and R120L (blue bars) hDAAO variants. The activity was assayed at 0.3 or 1 mM D-Ser and at three different FAD concentrations: 0, 2.5 or 40 μM. Activity values are reported as the mean ± SD (*n* = 3).

In order to assess the effect of the kinetic properties of R120 hDAAO variants on the oxidation of D-Ser under conditions similar to the physiological ones, the enzymatic activity was assayed on 0.3 or 1 mM D-Ser in the absence of free FAD or in the presence of 2.5 μM (corresponding to the maximal estimated concentration in the brain) or 40 μM FAD (a saturating cofactor concentration) (Cappelletti et al., 2015). In all cases, the conditions affected the activity only slightly (Figure 3). At low D-Ser and FAD concentrations higher activity is measured for the R120L variant, while at 1 mM D-Ser and 40 μM FAD, the wild-type hDAAO is the most active variant.

### Ligand and FAD-Binding

The interaction of R120 hDAAO variants with the classical inhibitor benzoate and with CBIO, the prototype of the new class of hDAAO inhibitors, was investigated following the protein fluorescence signal. Similar to what was observed for the wild-type hDAAO (Murtas et al., 2017), benzoate binding is a biphasic process for R120 variants. The main change observed was a ~3-fold higher K_d_ for the second phase of binding for the R120E variant, i.e., ~8 vs. 2.6 mM for the wild-type hDAAO (Table 3). Differently from benzoate, the binding of CBIO is a monophasic process for wild-type and variants of hDAAO.

**Table 3:** Binding properties of R120E and R120L hDAAO variants compared to the wild-type enzyme.

**hDAAO variants**	**FAD (K**_****d****_, **μM)**		**Benzoate (K_**d**_, μM)**	**CBIO (K_**d**_, μM)**
	**Free form**	**Benzoate complex^**a**^**		
Wild-type^b^	0.43 ± 0.02^c^ [40%]	0.48 ± 0.02	0.33 ± 0.04^c^	0.24 ± 0.05
	≥30^d^ [60%]		2,590 ± 680^d^	
R120E	0.05 ± 0.01^c^ [65%]	0.61 ± 0.08^c^ [65%]	0.16 ± 0.01^c^	0.30 ± 0.05
	32 ± 3^d^ [35%]	≥ 30^d^ [35%]	7,985 ± 755^d^	
R120L	0.05 ± 0.01^c^ [60%]	0.07 ± 0.01^c^ [75%]	0.27 ± 0.12^c^	0.35 ± 0.04
	23.1 ± 1.0^d^ [40%]	≥20^d^ [25%]	6,205 ± 40^d^	

*K_d_ values were determined by monitoring the quenching of protein fluorescence. When the FAD binding process to the apoprotein moiety is biphasic, the amplitude of fluorescence change for each phase is reported in square parentheses*.

a *In presence of 70 μM benzoate*.

b *Murtas et al. (2017)*.

c *K_d_ values corresponding to the first saturation phase*.

d *K_d_ values corresponding to the second saturation phase*.

**Figure 4:**
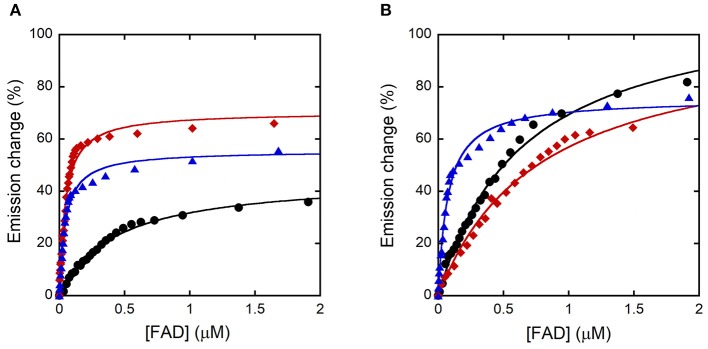
Analysis of FAD binding to hDAAO variants assessed as protein fluorescence quenching at ~340 nm. Wild-type (black), R120E (red), and R120L hDAAO (blue) apoprotein. Fit of the values, expressed as percentage of the total change, corresponding to: **(A)** the first saturation phase of the protein fluorescence change (up to 2 μM of cofactor) in the absence of benzoate; **(B)** in the presence of 70 μM of benzoate.

The K_d_ for FAD was estimated for the R120 hDAAO variants based on the quenching of the apoprotein fluorescence during FAD titration, both in the absence and presence of the active site ligand benzoate. Similar to the wild-type enzyme, FAD-binding is a biphasic process for both R120 variants in the absence of benzoate (Figure 4A): the fluorescence intensity change associated with the first phase accounts for 60–65% of the overall change. The main difference concerning the wild-type hDAAO is a 10-fold lower dissociation constant for the first phase (K_d1_) of cofactor binding to the apoprotein of R120 variants (Table 3). Significant alterations are also apparent when FAD binding to hDAAO apoprotein is performed in the presence of a saturating benzoate concentration (i.e., 70 μM, Figure 4B): this is a monophasic process for the wild-type hDAAO and a biphasic event for the R120 variants. Indeed, a tighter binding of the first process is apparent for the R120L variant only, while similar K_d1_ values are determined in the presence of benzoate for the wild-type and the R120E variants (Table 3).

Taken together, these findings indicate that R120 substitution modifies the interaction of hDAAO apoprotein with the flavin cofactor.

### Oligomeric State and Protein Stability

Wild-type hDAAO is a stable homodimer (Molla et al., 2006). R120 is located at the interface between monomers (Figure 1A) and, therefore, the oligomeric state of hDAAO R120E and R120L holoenzymes was investigated by gel-permeation chromatography. Both variants elute as a dimer in the 0.1–10 mg/mL protein concentration range (elution volume of ~13.7 mL, corresponding to a molecular mass of 80 kDa, not shown).

The stability of hDAAO secondary and tertiary structures was investigated by performing temperature-ramp experiments and analyzing the CD signal at 220 nm and the tryptophan fluorescence at 340 nm, respectively. The melting temperature values determined for the R120E and R120L variants are 3.5–5°C lower than those of wild-type hDAAO when the changes in tertiary structure are analyzed and ~10°C lower when the secondary structure stability is studied (Table 1).

### Subcellular Localization

**Figure 5:**
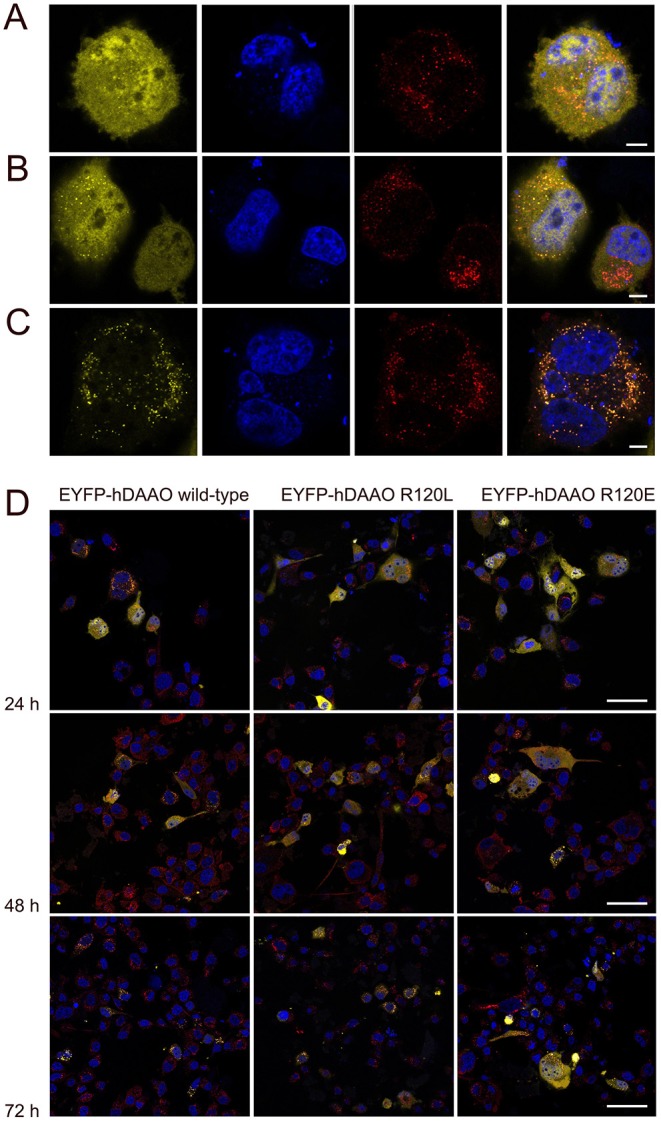
Confocal analysis of U87 cells expressing hDAAO variants. Cells were transiently transfected with the pEYFP-hDAAO-C3, pEYFP-hDAAO(R120L)-C3, and pEYFP-hDAAO(R120E)-C3 constructs. **(A–C)** Distribution of the overexpressed EYFP-hDAAO in different cellular compartments: **(A)** the EYFP-hDAAO signal (in yellow) is largely diffused in the cytosol and also clearly detectable within the nucleus (in blue). No overlapping with the peroxisomal stain (in red) is apparent; **(B)** the EYFP-hDAAO fluorescence is still present in the cytosol (and in the nucleus, in this case), but a punctuate distribution overlapping to the peroxisomal stain is evident; **(C)** the EYFP-hDAAO is fully compartmentalized, as indicated by the almost complete overlapping of the corresponding signal with peroxisomal stain. Note that no EYFP-hDAAO is detectable in the nucleus. **(D)** Time course after transfection of the subcellular distribution of EYFP-hDAAO variants in U87 transfected cells. Scale bars: **(A–C)** 5 μm; **(D)** 50 μm.

**Figure 6:**
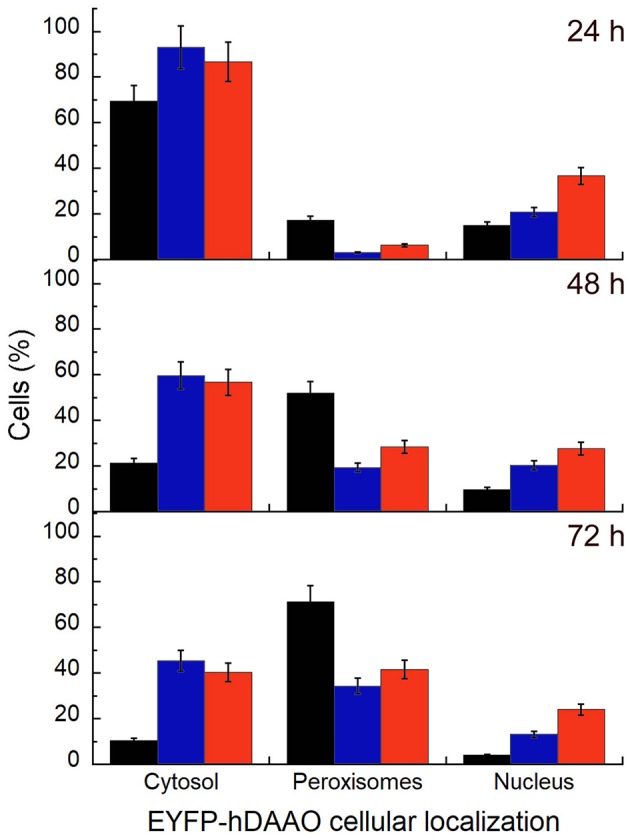
Time course of the subcellular distribution of EYFP-hDAAO variants in transiently transfected U87 cells investigated by immunostaining and confocal analysis. The cells were fixed at different times after transfection (24, 48, and 72 h) and immunostained with an anti-PMP70 antibody to specifically label the peroxisomes. Nuclei were counterstained using the red fluorescent probe DRAQ5™ and counted to obtain the total number of cells. Data are reported as the fraction of transfected cells (at different times after transfection) in which the EYFP-hDAAO variants signal is largely observed in the cytosol, or is completely compartmentalized within the peroxisomes, or is detected in the nucleus. Black bars, EYFP-hDAAO wild-type; blue bars, EYFP-hDAAO R120L; red bars, EYFP-hDAAO R120E. Values represent mean ± SEM.

U87 glioblastoma cells were transiently transfected with a pEYFP-hDAAO-C3 plasmid encoding for EYFP-hDAAO chimeric proteins harboring R, E, or L at position 120 of the flavoenzyme. The presence of the fluorescence tag at the N-terminal end was previously demonstrated to not alter the activity, subcellular distribution, or degradation pathway of wild-type hDAAO (Sacchi et al., 2008, 2011; Li et al., 2013). A similar amount of transfected cells (expressing EYFP-hDAAO protein) was obtained for all three constructs (Table 4). The percentage of cells showing a cytosolic, peroxisomal, and nuclear localization of the flavoenzyme was determined by comparing the yellow fluorescence signal of EYFP-hDAAO variants with those of the markers PMP-70 (for peroxisomes) and DRAQ5™ dye (for nuclei, Figure 5). The amount of cytosolic wild-type EYFP-hDAAO decreased with time after transfection (28% of the signal is cytosolic at 72 h vs. 94% at 24 h), as did the amount of nuclear signal (Table 4 and Figure 6). The presence of glutamate at position 120 of hDAAO to mimic a phosphorylation state favors the mistargeting by increasing the number of cells with an EYFP nuclear signal as compared to wild-type hDAAO. This is accompanied by a lower percentage of cells displaying a peroxisomal signal for the EYFP-tagged protein. Introducing an uncharged residue at position 120 (R120L variant) also negatively affects peroxisomal targeting and modifies, to a limited extent, the percentage of cells with an EYFP-hDAAO nuclear signal as compared to the cells expressing the wild-type hDAAO (Figure 5). The substitution of R120 also appears to increase the propensity of hDAAO to form cytosolic aggregates (Table 4). Notably, in all cases, the cells showing nuclear localization of hDAAO also possess a signal largely diffused in the cytosol.

**Table 4:** Confocal analysis of the cellular distribution of hDAAO variants in U87 cells transiently transfected with different pEYFP-hDAAO expression constructs.

**hDAAO variants**	**Hours after transfection**	**Cells number**	**T (%)**	**hDAAO cellular distribution (% of transfected cells)**				
				**C**	**C/P**	**P**	**N**	**A**
Wild-type	24	551	24.3	69.4	13.4	17.2	14.4	18.7
	48	599	17.4	21.2	26.9	51.9	9.6	15.4
	72	781	16.0	10.4	18.4	71.2	4.0	5.6
R120L	24	628	20.7	93.1	3.8	3.1	20.8	41.5
	48	497	24.9	59.7	21.0	19.4	20.2	22.6
	72	511	19.4	45.5	21.2	34.3	13.1	35.6
R120E	24	515	24.9	86.7	7.8	6.3	36.7	29.7
	48	549	24.4	56.7	14.9	28.4	27.6	31.3
	72	801	19.2	40.3	18.2	41.6	24.0	18.2

*The cells were fixed at different times after transfection and peroxisomes were immunostained using an anti-PMP70 antibody. The total number of cells in each field was determined by counting the nuclei, counterstained using the DRAQ5™ far red fluorescent dye. T, transfected cells; C, cytosol; C/P, mixed, cytosol and peroxisomes; P, peroxisomes; N, nucleus; A, large cytosolic aggregates detected*.

The number of viable cells following transfection is similar for wild-type and R120 hDAAO variants, indicating that mistargeting does not affect cell viability.

## Discussion

The elucidation of the structure-function relationships in hDAAO represents a way to deep inside the modulation of the D-serine level in the human brain. Here we studied two enzyme variants at position 120. From a biochemical point of view, substituting R120 in hDAAO slightly alters protein conformation, kinetic properties, and ligand binding, while the dimeric quaternary structure is unchanged. The tertiary structure is largely preserved: only for the R120E hDAAO does alteration in the near-UV CD and fluorescence spectra suggest that the orientation of aromatic residues has changed and is coupled to a decrease in the α-helix content. Indeed, both the R120E and R120L variants show a decrease in T_m_, which is more evident following the signal related to the secondary structure (Table 1): the substitution of R120 likely alters the electrostatic interactions with the facing residues belonging to α3 and α6 and the loop connecting α5 and β4 on the other monomer (Figure 1C). Notably, the substitution of R120 in hDAAO does not alter the affinity for the classical inhibitor benzoate (resulting in a biphasic binding process) and for CBIO (showing a monophasic process; Table 3). This result excludes a connection between R120 and the ligand-binding process, especially related to the second putative benzoate binding site.

Concerning the apparent kinetic properties, while both of the variants at position 120 possess a higher kinetic efficiency for D-Ala and D-Cys due to a higher *k*_*cat*_ than the wild-type enzyme, a similar kinetic efficiency value is apparent on D-Ser as the increase in maximal activity is counteracted by an increased K_m_ value. D-Ser is the physiological substrate of hDAAO in the brain: the low kinetic efficiency of the human flavoenzyme on this substrate seems to be evolved to control its cerebral level without resulting in a drastic decrease that could negatively affect NMDAr functionality (Caldinelli et al., 2009, 2010; Murtas et al., 2017; Pollegioni et al., 2018). The possibility that hDAAO kinetic properties could be affected by post-translational modification processes is a topic that deserves further investigations.

The most relevant change in hDAAO following the substitution of R120 is the strongest interaction with the flavin cofactor. The presence in solution of two apoprotein forms showing a different affinity for FAD is still evident for R120 variants, but the most avid form (accounting for ~ 60-65% of the total change in protein fluorescence following FAD-binding) possesses a 10-fold lower K_d_ than the corresponding form of wild-type hDAAO (Table 3). This could be significant at the cellular level as wild-type hDAAO at physiological FAD concentration (~2 μM) is in equilibrium between the apoprotein inactive form and the active holoenzyme. In any case, the activity values for wild-type and R120 hDAAO variants were quite similar at low D-Ser and FAD concentrations, resembling the physiological conditions (Figure 3). Furthermore, we previously reported that benzoate binding shifts the hDAAO apoprotein into the single conformation for FAD more avidly (Caldinelli et al., 2009, 2010; Murtas et al., 2017). This ligand-induced conformation change is abolished for R120 variants: FAD-binding to hDAAO variants is biphasic both in the absence and in the presence of the active-site ligand.

The properties of W209R hDAAO, corresponding to the SNP rs11347906 (Cappelletti et al., 2015), resemble those of R120 variants: the residue W209 is also located at the monomer-monomer interface, is part of the second binding site identified by Kohiki et al. (2017), and its substitution results in an increased activity on D-Ala, a 4-fold tighter FAD-binding with no change in ligand-binding properties (Cappelletti et al., 2015). The similarity in biochemical properties between R120E/L and W209R hDAAO variants suggests that the reported alterations are due to the structure-function relationships of this specific region, which is close to the interface between monomers.

Concerning the investigation of hDAAO subcellular localization, and since phosphorylation of arginine is unusual, we substituted R120 with glutamate (to mimic the active NTS sequence) or a leucine (to eliminate the positive charge). Our studies based on U87 glioblastoma cells transiently transfected for hDAAO variants show that substitution of R120 alters the subcellular localization of the flavoenzyme; the increase in cytosolic localization, observed at all times after translocation, favors nuclear targeting, especially for the R120E hDAAO (Figures 5, 6). Indeed, the wild-type enzyme is also mistargeted to the nucleus, suggesting that it is a usual process for hDAAO. Nuclear passive diffusion promotes the transport of proteins of <40 kDa (corresponding to the size of DAAO monomer) although proteins at higher mass also accumulate in the nucleus (Luks et al., 2017b). R120 is located at the protein interface between monomers and electrostatically interacts with D73 on the opposite chain (Figure 1C). The substitution of the arginine in position 120 does not affect the generation of a soluble, dimeric holoenzyme: the altered subcellular localization of R120 hDAAO variants does not arise from a change in molecular mass. Finally, the expression of catalytically active hDAAO variants at position 120 does not alter the viability of U87 transfected cells. This is significant and can be explained in two different ways: (i) mistargeted (cytosolic and nuclear) hDAAO is inactive; (ii) no hydrogen peroxide is generated by mistargeted hDAAO because of the absence of its substrate, i.e., the D-amino acids.

Results from mutagenesis at position 120 and previous studies on the W209R hDAAO variant (Cappelletti et al., 2015) indicate that single point substitutions at the monomer-monomer interface affect biochemical properties, especially the affinity for FAD, but do not affect ligand binding. Indeed, our results reveal that mistargeting to the nucleus is an innate process, as it is apparent for the wild-type enzyme too (Table 4), which does not affect cell viability. Whether hDAAO mistargeting is involved in physiological processes or related to specific pathological states is still unknown (Pollegioni et al., 2018).

## Data Availability Statement

The datasets generated for this study are available on request to the corresponding author.

## Author Contributions

LP and SS conceived the project. GM and SS performed the experiments. All the authors analyzed the data and wrote the paper.

### Conflict of Interest

The authors declare that the research was conducted in the absence of any commercial or financial relationships that could be construed as a potential conflict of interest.

## Abbreviations

CBIO: 6-chloro-benzo(*d*)isoxazol-3-ol
CD: circular dichroism
DAAO: D-amino acid oxidase (EC 1.4.3.3)
D-Ala: D-alanine
D-Cys: D-cysteine
D-Ser: D-serine
EYFP: enhanced yellow fluorescent protein
hDAAO: human D-amino acid oxidase
NMDAr: N-methyl-D-aspartate subtype of glutamate receptor
NTS: nuclear translocation signal.
